# Lack of validation of genetic variants associated with anti–tumor necrosis factor therapy response in rheumatoid arthritis: a genome-wide association study replication and meta-analysis

**DOI:** 10.1186/ar4504

**Published:** 2014-03-11

**Authors:** Ana Márquez, Aida Ferreiro-Iglesias, Cristina L Dávila-Fajardo, Ariana Montes, Dora Pascual-Salcedo, Eva Perez-Pampin, Manuel J Moreno-Ramos, Rosa García-Portales, Federico Navarro, Virginia Moreira, César Magro, Rafael Caliz, Miguel Angel Ferrer, Juan José Alegre-Sancho, Beatriz Joven, Patricia Carreira, Alejandro Balsa, Yiannis Vasilopoulos, Theologia Sarafidou, José Cabeza-Barrera, Javier Narvaez, Enrique Raya, Juan D Cañete, Antonio Fernández-Nebro, María del Carmen Ordóñez, Arturo R de la Serna, Berta Magallares, Juan J Gomez-Reino, Antonio González, Javier Martín

**Affiliations:** 1 Instituto de Parasitología y Biomedicina López-Neyra, Consejo Superior de Investigaciones Científicas (CSIC), Avenida del Conocimiento s/n, 18016-Armilla, Granada, Spain; 2 Laboratorio Investigación 10 and Rheumatology Unit, Instituto de Investigación Sanitaria-Hospital Clínico Universitario de Santiago, Travesia Choupana s/n 15706-Santiago de Compostela, Spain; 3 Department of Clinical Pharmacy, Hospital Universitario San Cecilio, Instituto de Investigación Biosanitaria de Granada (IBIG), Avenida Doctor Oloriz 16, 18012-Granada, Spain; 4 Department of Immunology, Instituto de Investigación Sanitaria del Hospital Universitario La Paz (IdiPAZ), Research Network in Inflammation and Rheumatic Diseases (RIER), Hospital La Paz, Paseo de la Castellana, 261, 28046-Madrid, Spain; 5 Department of Rheumatology, Hospital Virgen de la Arrixaca, Ctra. Madrid-Cartagena s/n, 30120-El Palmar Murcia, Spain; 6 Department of Rheumatology, Hospital Virgen de la Victoria, Campus de Teatinos s/n, 29010-Málaga, Spain; 7 Rheumatology Unit, Hospital Universitario Virgen Macarena, Avenida Dr. Fedriani 3, 41007-Sevilla, Spain; 8 Department of Rheumatology, Hospital Clínico San Cecilio, Avenida Doctor Oloriz 16, 18012-Granada, Spain; 9 Rheumatology Unit, Hospital Universitario Virgen de las Nieves, Avenida de las Fuerzas Armadas 2, 18014-Granada, Spain; 10 Department of Rheumatology, Hospital Doctor Peset, Avenida de Gaspar Aguilar 90, 46017-Valencia, Spain; 11 Department of Rheumatology, Instituto de Investigación I+12, Research Network in Inflammation and Rheumatic Diseases (RIER), Hospital Universitario 12 de Octubre, Avenida de Córdoba s/n, 28041-Madrid, Spain; 12 Department of Rheumatology, Instituto de Investigación Sanitaria del Hospital Universitario La Paz (IdiPAZ), Research Network in Inflammation and Rheumatic Diseases (RIER), Paseo de la Castellana, 261, 28046-Madrid, Spain; 13 Department of Biochemistry and Biotechnology, University of Thessaly, 26 Ploutonos & Aiolou Str., 41221-Larissa, Greece; 14 Rheumatology Department, Hospital Universitario de Bellvitge, Feixa Llarga s/n, 08907-L'Hospitalet de Llobregat, Barcelona, Spain; 15 Rheumatology Department, Hospital Clinic and Institut d’Investigacions Biomèdiques August Pi I Sunyer (IDIBAPS), Calle Villarroel 170, 08036-Barcelona, Spain; 16 Unidad de Gestión Clínica (UGC) Rheumatology, Hospital Regional Universitario de Málaga, Instituto de Investigación Biomédica de Málaga (IBIMA), Avenida Carlos Haya s/n, 29010-Málaga, Spain; 17 Rheumatology Unit, Hospital Santa Creu e San Pau, Carrer de Sant Quintí 89, 08026-Barcelona, Spain; 18 Department of Medicine, University of Santiago de Compostela, Rúa de San Francisco s/n, 15782-Santiago de Compostela, Spain

## Abstract

**Introduction:**

In this study, our aim was to elucidate the role of four polymorphisms identified in a prior large genome-wide association study (GWAS) in which the investigators analyzed the responses of patients with rheumatoid arthritis (RA) to treatment with tumor necrosis factor inhibitors (TNFi). The authors of that study reported that the four genetic variants were significantly associated*.* However, none of the associations reached GWAS significance, and two subsequent studies failed to replicate these associations.

**Methods:**

The four polymorphisms (rs12081765, rs1532269, rs17301249 and rs7305646) were genotyped in a total of 634 TNFi-treated RA patients of Spanish Caucasian origin. Four outcomes were evaluated: changes in the Disease Activity Score in 28 joints (DAS28) after 6 and 12 months of treatment and classification according to the European League Against Rheumatism (EULAR) response criteria at the same time points. Association with DAS28 changes was assessed by linear regression using an additive genetic model. Contingency tables of genotype and allele frequencies between EULAR responder and nonresponder patients were compared. In addition, we combined our data with those of previously reported studies in a meta-analysis including 2,998 RA patients.

**Results:**

None of the four genetic variants showed an association with response to TNFi in any of the four outcomes analyzed in our Spanish patients. In addition, only rs1532269 yielded a suggestive association (*P* = 0.0033) with the response to TNFi when available data from previous studies were combined in the meta-analysis.

**Conclusion:**

Our data suggest that the rs12081765, rs1532269, rs17301249 and rs7305646 genetic variants do not have a role as genetic predictors of TNFi treatment outcomes.

## Introduction

Rheumatoid arthritis (RA) is a systemic autoimmune disease characterized by chronic inflammation of the synovial joints resulting in joint destruction, polyarthritis and functional disability. This inflammatory condition affects approximately 1% of the Caucasian population, making it a significant cause of comorbidity and mortality [1].

In recent years, the use of tumor necrosis factor inhibitors (TNFi) has resulted in an improvement in the treatment of RA patients by reducing both inflammation and joint damage [2-4], and their clinical use has become widespread. However, a percentage of patients do not respond adequately to this therapy; therefore, the current use of these agents is based on a trial-and-error approach [5,6]. Given the adverse effects and the high cost of this type of therapy, the establishment of pharmacogenetic markers to predict the response to TNFi treatment is a highly desirable goal.

Recently, researchers in pharmacogenetic studies have reported several genetic variants associated with clinical response to treatment with TNFi [7-11]. However, to date, only the *PTPRC* and *PDE3A-SLCO1C1 loci* have been associated in more than a single study [12-14].

In 2011, Plant *et al*. [8] performed a genome-wide association study (GWAS) in a large number of RA patients from the United Kingdom treated with TNFi. These investigators used a three-stage study design. The meta-analysis combining stages 1, 2 and 3 cohorts yielded four single-nucleotide polymorphisms (SNPs) putatively associated with the TNFi response at 6 months, although these associations did not reach the GWAS significance level. Two of the genetic variants mapped within genes, PDZ domain-containing 2 (*PDZD2*) and eyes absent homolog 4 (*EYA4*), and two polymorphisms mapped to intergenic regions on chromosomes 1 and 12. However, two subsequent GWASs conducted in European RA patients, whose treatment response was evaluated at 14 weeks, failed to replicate association with any of the four *loci*[9,10].

The aim of our study was to assess the role of the four genetic variants identified by Plant *et al.*[8] with regard to their association with response to TNFi using a large number of RA Spanish patients, as well as to conduct a meta-analysis including previous data.

## Methods

### Patients

Two sets of RA patients of Spanish ancestry treated with TNFi (infliximab, adalimumab and etanercept) were included in the study. Collection 1 comprised 438 patients, and collection 2 included 196 patients. All patients were classified according to the 1987 American Rheumatism Association criteria [15]. Informed written consent from all participants and approval from the local ethical committees (Comité Ético de Investigación Clínica de Galicia and Comité de Bioética del Consejo Superior de Investigaciones Científicas) were obtained in accordance with the tenets of the Declaration of Helsinki. The characteristics of the patients enrolled in this study are shown in Table 1.

**Table 1:** **Baseline characteristics of the rheumatoid arthritis patients**^**a**^

**Baseline characteristics**	**Collection 1 (*N *** **= 438)**	**Collection 2 (*N *** **= 196)**
Age, mean ± SD years	61.0 ± 12.04	56.3 ± 14.77
Age at diagnosis, mean ± SD years	44.95 ± 13.03	42.99 ± 13.69
Females, *n* (%)	365 (83.3)	160 (81.6)
Disease duration, mean ± SD years	16.45 ± 8.34	12.93 ± 8.47
Rheumatoid factor–positive, *n* (%)	340 (77.8)	141 (71.94)
Anti-CCP-positive, *n* (%)	250 (69.6)^b^	125 (73.1)^c^
Smoking status, *n* (%)		
Ever-smoker	51 (16.0)^b^	20 (13.9)^c^
Never-smoker	267 (84.0)^b^	124 (86.1)^c^
Health status, mean ± SD		
DAS28 at baseline	5.86 ± 1.12	5.36 ± 1.13
Treatment, *n* (%)		
Concurrent DMARDs	252 (92.6)^b^	159 (81.1)
Previous biologic agents	0 (0)	14 (10.2)^c^
Anti-TNF drugs, *n* (%)		
Infliximab	245 (55.9)	62 (31.6)
Etanercept	113 (25.8)	21 (10.7)
Adalimumab	80 (18.3)	113 (57.7)
EULAR-defined response at 6 months, *n* (%)		
Responders	337 (80.4)	167 (85.2)
Nonresponders	82 (19.6)	29 (14.8)
EULAR-defined response at 12 months, *n* (%)		
Responders	259 (82.5)	118 (88.1)
Nonresponders	55 (17.5)	16 (11.9)

^a^Anti-CCP, Anti–cyclic citrullinated peptide antibody; DAS28, Disease Activity Score in 28 joints; DMARD, Disease-modifying antirheumatic drug; EULAR, European League Against Rheumatism; TNF, Tumor necrosis factor. ^b^Data are from <85% of the patients: 359 for anti-CCP status, 318 for smoking status and 272 for concurrent DMARD treatment. ^c^Data are from <70% of the patients: 171 for anti-CCP status, 144 for smoking status and 137 for previous biologic agent treatment.

### Treatment outcomes

The Disease Activity Score in 28 joints (DAS28) was measured at baseline and at 6 and 12 months after the first TNFi infusion. Two scales were considered to assess the efficacy of the TNFi therapy. First, the absolute change in DAS28 (∆DAS28 = DAS28_end_ – DAS28_baseline_) at 6 and 12 months of follow-up. Second, patients were classified as responders (good and moderate) or nonresponders at 6 and 12 months according to the European League Against Rheumatism (EULAR) response criteria [16].

### Genotyping

Genomic DNA was extracted from peripheral white blood cells or saliva using standard procedures. Four SNPs—rs1532269 and rs17301249, intronic polymorphisms mapped within *PDZD2* and *EYA4*, respectively, and rs12081765 and rs7305646 located at intergenic regions on chromosomes 1 and 12, respectively—were genotyped using a single-base extension technology (SNaPshot Multiplex Kit; Applied Biosystems, Foster City, CA, USA) in a multiplex PCR experiment (KAPA2G Fast HotStart; Kapa Biosystems, Wilmington, MA, USA) in collection 1 and using TaqMan allelic discrimination assays on a 7900HT Fast Real-Time PCR System, both purchased from Applied Biosystems, in collection 2. A deviation from Hardy–Weinberg equilibrium (HWE) was detected for the rs1532269 polymorphism in collection 2, so that SNP was genotyped in this sample set using the same methodology used for collection 1.

### Statistical analysis

Power calculations were performed using Quanto version 1.2.4 software [17]. All SNPs were tested for deviations from HWE. The association between SNPs and responses to TNFi was evaluated in two ways. In the first method, linear regression analysis using ∆DAS28 as the continuous dependent variable was performed under an additive genetic model using Plink version 1.07 statistical software [18]. A *t*-test was used to identify polymorphisms associated with the response. In the second method, genotype and allele frequencies between EULAR-defined responder and nonresponder patients were compared. Plink was used to create 2 × 2 or 2 × 3 contingency tables and to perform a *χ*^2^ test and/or Fisher’s exact test. Odds ratios (ORs) and 95% confidence intervals (CIs) were calculated according to Woolf’s method. Because our present study is a replication study, no correction was applied to the obtained *P*-values when TNFi response was evaluated at 6 months. In the analyses involving the TNFi efficacy at 12 months, however, *P*-values were corrected by using the Benjamini–Hochberg step-up procedure to control for false discovery rates (FDRs) in multiple testing [19]. The results were considered statistically significant when *P*-values were lower than 0.05.

Clinical variables previously identified as being independent predictors of efficacy of TNFi, including age, gender, smoking status, rheumatoid factor status, anti–cyclic citrullinated peptide antibody (anti-CCP) status, DAS28 at baseline, concurrent and previous treatment and TNFi, were assessed for association with treatment response in a multivariate regression analysis using *STATISTICA* version 7.0 software (StatSoft, Tulsa, OK, USA) and Plink software in collections 1 and 2, respectively. Only baseline DAS28, gender and TNFi were associated with the efficacy of the therapy. Accordingly, analyses were adjusted for these three variables.

The analysis of the combined data from our study and the previous reports [8-10] was performed using Plink. Heterogeneity between studies was assessed using Cochran’s *Q* and *I*^2^ statistics [20]. Pooled analyses were performed by using the Mantel–Haenszel test under the fixed-effects model or the DerSimonian–Laird test under the random-effects model when heterogeneity was detected. The results were considered to be statistically significant when *P*-values were lower than 5 × 10^5.00E−08^.

## Results

All of the four studied polymorphisms conformed to HWE expectations (*P* > 0.01). The genotyping success rate was higher than 95%.

### Replication study

First, we analyzed the association between the four tested polymorphisms and the efficacy of the TNFi therapy in the 438 RA patients of Spanish Caucasian origin in collection 1. As shown in Table 2, in the linear regression analysis using ∆DAS28, none of the analyzed genetic variants were associated with the clinical response at 6 months (*P* = 0.570, *P* = 0.831, *P* = 0.181 and *P* = 0.244 for rs12081765, rs1532269, rs17301249 and rs7305646, respectively) or at 12 months (*P* = 0.716, *P* = 0.647, *P* = 0.416 and *P* = 0.182 for rs12081765, rs1532269, rs17301249 and rs7305646, respectively). Likewise, when allele frequencies were compared between responder and nonresponder patients, no association with the EULAR-defined response at 6 or 12 months was observed for any of the analyzed polymorphisms (see Additional file 1: Tables S1 and S2).

**Table 2:** **Association of the four single-nucleotide polymorphisms with changes in Disease Activity Score in 28 joints at 6 and 12 months in Spanish rheumatoid arthritis patients**^**a**^

			**6 months**						**12 months**					
			**Collection 1 (*N *** **= 419)**			**Collection 2 (*N *** **= 193)**			**Collection 1 (*N *** **= 314)**			**Collection 2 (*N *** **= 134)**		
**SNP**	***Locus***	**1/2**	**MAF**	***P*-value**^**b**^	**β**^**b**^	**MAF**	***P*-value**^**b**^	**β**^**b**^	**MAF**	***P*-value**^**b**^	**β**^**b**^	**MAF**	***P*-value**^**b**^	**β**^**b**^
rs12081765	Intergenic	A/G	0.415	0.570	0.055	0.458	0.995	0.001	0.411	0.716	0.043	0.408	0.677	0.062
rs1532269	*PDZ2D*	C/G	0.375	0.831	0.020	0.418	0.830	0.022	0.400	0.647	0.051	0.420	0.022	0.335
rs17301249	*EYA4*	C/G	0.121	0.181	−0.190	0.132	0.458	0.120	0.131	0.416	−0.137	0.154	0.529	0.135
rs7305646	Intergenic	T/C	0.474	0.244	−0.117	0.487	0.661	−0.049	0.486	0.182	−0.161	0.473	0.554	−0.093

^a^1, Minor allele, 2, Major allele; MAF, Minor allele frequency; SNP, Single-nucleotide polymorphism. ^b^Adjusted for gender, anti–tumor necrosis factor treatment and Disease Activity Score in 28 joints at baseline.

In the subsequent analysis in collection 2, none of the tested polymorphisms showed an association with ∆DAS28 at 6 months (Table 2) (*P* = 0.995, *P* = 0.830, *P* = 0.458 and *P* = 0.661 for rs12081765, rs1532269, rs17301249 and rs7305646, respectively) or in the stratified analysis according to the EULAR-defined response (see Additional file 1: Table S1). When TNFi efficacy was evaluated at 12 months, the rs1532269 polymorphism showed an association with ∆DAS28 at that time point (Table 2) (*P* = 0.022, β = 0.335); however, statistical significance was lost after correction using the Benjamini–Hochberg step-up procedure for FDR (*P*_FDR_ = 0.087). No association of this SNP with the EULAR-defined response at the 12-month time point was observed (see Additional file 1: Table S2).

No heterogeneity was observed (*P* > 0.1 by Cochran’s *Q*-statistic) before we performed the meta-analysis of the two Spanish collections. No association between rs12081765, rs1532269, rs17301249 and rs7305646 and the efficacy of the TNFi therapy was evident in this pooled analysis for any of the outcomes considered as measured by ∆DAS28 (Table 3) and EULAR-defined response (see Additional file 1: Table S3).

**Table 3:** Pooled analysis of the tested polymorphisms in the two Spanish cohorts

		**Meta-analysis**			
		**6 months**		**12 months**	
**Single-nucleotide polymorphism**	***Locus***	***P*-value**	**β**	***P*-value**	**β**
rs12081765	Intergenic	0.677	0.029	0.586	0.050
rs1532269	*PDZ2D*	0.762	0.021	0.074	0.158
rs17301249	*EYA4*	0.607	−0.055	0.8041	−0.033
rs7305646	Intergenic	0.246	−0.086	0.1549	−0.136

### Meta-analysis of all available studies

We combined our data with the results of three previous studies in order to assess the global status of the four polymorphisms [8-10]. Results corresponding to ∆DAS28 at 14 weeks from two of the studies [9,10] were combined with results at 24 weeks from the other two studies ([8] and present meta-analysis). A total of 2,998 RA patients were included in the meta-analysis, which had >90% power to detect a difference of ≥0.6 units in ∆DAS28 at the GWAS significance threshold (*P* ≤ 5.0E-08) for allele frequencies ≥10%. Only one of the polymorphisms, rs1532269, showed a suggestive association (fixed-effects model: *P* = 0.0033, β = 0.107) (Table 4), although it did not reach the GWAS significance level. The other three were not associated with ∆DAS28 at 3 to 6 months (random-effects model: *P* = 0.102, β = 0.068; *P* = 0.063, β = −0.138; and *P* = 0.085, β = −0.095, for rs12081765, rs17301249 and rs7305646, respectively) (Table 4). When data derived from the four studies were pooled, heterogeneity for the rs12081765, rs17301249 and rs7305646 variants was evident (Cochran’s *Q*-statistic *P* < 0.05, *I*^2^ > 40%).

**Table 4:** **Meta-analysis of association of four single-nucleotide polymorphisms with changes in Disease Activity Score in 28 joints in all available studies**^**a**^

		**rs12081765**			**rs1532269**			**rs17301249**			**rs7305646**		
**Study**		**MAF**	***P*-value**	**β**	**MAF**	***P*-value**	**β**	**MAF**	***P*-value**	**β**	**MAF**	***P*-value**	**β**
Plant *et al*. [8]	Cohort 1 (*n* = 566)	0.43	7.52E-04	0.29	0.37	7.11E-04	0.3	0.20	3.37E-04	−0.38	0.47	9.16E-04	−0.28
	Cohort 2 (*n* = 379)	0.46	0.062	0.19	0.35	0.079	0.19	0.19	0.045	−0.24	0.49	0.049	−0.21
	Cohort 3 (*n* = 341)	0.47	0.712	0.04	0.34	0.701	0.04	0.19	0.261	−0.14	0.50	0.292	−0.11
Krintel *et al*. [9]	Copenhagen cohort (*n* = 196)	0.47	0.940	0.008	0.36	0.360	0.116	0.18	0.570	0.89	0.48	0.740	0.042
Umicevic Mirkov *et al*. [10]	Dutch stage 1 cohort (*n* = 882)	0.39	0.730	−0.024	0.43	0.920	0.051	0.18	0.360	−0.05	0.50	0.390	0.05
Present study	Collection 1 (*n* = 438)	0.42	0.570	0.055	0.38	0.831	0.020	0.12	0.181	−0.190	0.47	0.244	−0.117
	Collection 2 (*n* = 196)	0.46	0.995	0.001	0.42	0.830	0.022	0.13	0.458	0.120	0.49	0.661	−0.049
Meta-analysis	*N* = 2,998		0.102	0.068		0.0033	0.107		0.063	−0.138		0.085	−0.095

^**a**^MAF, Minor allele frequency.

## Discussion

Our results make it unlikely that any of the four polymorphisms identified by Plant *et al*. could be used as genetic predictors of TNFi treatment outcomes, because they were not associated in our large Spanish RA study. This lack of association represents a very relevant finding because, to the best of our knowledge, our present study is the first in which the association of these SNPs was analyzed with the same treatment outcomes used for their identification. In addition, the combined analysis with the three previous studies included in our meta-analysis [8-10] showed only a suggestive association of one of the four polymorphisms (even weaker than that reported in the study by Plant *et al*. [8]). These findings seem to exclude effects of sufficient magnitude to be useful in predicting response to treatment.

The lack of replication in our study could be ascribed to multiple differences between studies. It is commonly impossible to identify one of them as being more relevant than the others. Genetic differences between populations are an unlikely explanation of the results, given that the allele frequencies of the four tested polymorphisms were very similar between studies. Clinical differences between the patients with RA included in the different reports are possible and difficult to exclude. In this regard, it has already been mentioned that Plant *et al*. [8] evaluated the response to TNFi at 6 months, whereas the two subsequent studies used the response at 14 weeks. However, this difference does not apply to our study in which evaluation at 6 months evidenced negative results. A common cause of discrepant results is the overestimation of the true effect in the discovery study. This phenomenon has been characterized as the “winner’s curse,” and it has been very prevalent in genetic association studies [21]. It should be noted that the four SNPs studied by Plant *et al*. [8] showed the highest effects in the discovery cohort (which was the only one with a clear association between these four polymorphisms and the clinical response), whereas the three replication studies showed lower effect sizes (β-values less different from zero), thus supporting this possibility. Indeed, significant heterogeneity between studies was observed in the meta-analysis of three of the four analyzed genetic variants. Interestingly, this heterogeneity disappeared when the discovery cohort of Plant *et al*. was removed [8]. Therefore, variables other than the presence of the four SNPs considered herein could have influenced the efficacy of TNFi in this cohort, accounting for its singularity.

Other GWASs of responses to TNFi treatment in RA have been published [7,9-11]. This approach represents an important step forward in the understanding of the influence of genetic variability on the efficacy of this therapy. Only one of the observed associations has been found to reach the GWAS statistical significance level, however, and only after combination with data derived from replication studies [12]. This highlights the important role of validation studies in determining the status of the remaining GWAS findings. It is to be expected that these combined efforts will produce useful insights.

## Conclusions

The association of four polymorphisms (rs12081765, rs1532269, rs17301249 and rs7305646) previously identified as being associated with TNFi treatment response was not confirmed in the present study. Our results indicate that these four genetic variants are not useful predictors of response to TNFi in patients with RA.

## Abbreviations

∆DAS28: Absolute change in Disease Activity Score in 28 joints; ACR: American College of Rheumatology; CCP: Cyclic citrullinated peptide; CI: Confidence interval; DAS28: Disease Activity Score in 28 joints; EULAR: European League Against Rheumatism; GWAS: Genome-wide association study; RA: Rheumatoid arthritis; SNP: Single-nucleotide polymorphism; TNF: Tumor necrosis factor.

## Competing interests

The work done at Consejo Superior de Investigaciones Científicas (CSIC) was supported by AbbVie Pharmaceuticals and partially by *Cooperative Research Thematic Network* (RETICS) programs RD08/0075/0011, RD12/0009/0004, RD12/0009/0012 and RD12/0009/0016 (RIER); “Instituto de Salud Carlos III” (ISCIII, Health Ministry, Madrid, Spain); and grants from the European Innovative Medicines Initiative (IMI) Be the Cure for Arthritis (BTCure) program. The work done in Santiago de Compostela was funded by Fondo de Investigación Sanitaria from ISCIII, (Health Ministry, Madrid, Spain), grants PI11/01048, PI12/01909, RD08/0075/0019 and RD12/0009/0008, which are partially financed by the European Regional Development Fund of the European Union. The sponsors had no role in the design of the study; in the collection, analysis or interpretation of data; or in the preparation, review or approval of the manuscript.

## Authors’ contributions

AMá, AFI, AG and JM were involved in the conception and design of the study. AMá, AFI, CLDF and AMo contributed to the analysis and interpretation of data. AMá drafted the manuscript. DPS, EPP, MJMR, RGP, FN, VM, CM, RC, MAF, JJAS, BJ, PC, AB, YV, TS, JCB, JN, ER, JDC, AFN, MCO, ARS, BM, JJGR and AG collected samples and participated in the analysis and interpretation of data. AFI, CLDF, DPS, EPP, MJMR, RGP, FN, VM, CM, RC, MAF, JJAS, BJ, PC, AB, YV, TS, JCB, JN, ER, JDC, AFN, MCO, ARS, BM, JJGR, AG and JM revised the manuscript draft critically. All authors read and approved the final manuscript.

## Supplementary Material

Additional file 1: Table S1 Association analysis of the four analyzed genetic variants with the European League Against Rheumatism (EULAR) response criteria at 6 months in the Spanish rheumatoid arthritis (RA) patients. **Table S2.** Association analysis of the four analyzed genetic variants with the European League Against Rheumatism (EULAR) response criteria at 12 months in the Spanish rheumatoid arthritis (RA) patients. **Table S3.** Meta-analysis of the four tested genetic variants in non-responder and responder rheumatoid arthritis (RA) patients from the two Spanish collections. Click here for file
