# Supplementary material for: Lack of validation of genetic variants associated with anti–tumor necrosis factor therapy response in rheumatoid arthritis: a genome-wide association study replication and meta-analysis
Source: Arthritis Res Ther. 2014 Mar 11;16(2):R66. doi: 10.1186/ar4504 (PMC4060376; doi:10.1186/ar4504)
Supplement: Additional file 1: Table S1 — Association analysis of the four analyzed genetic variants with the European League Against Rheumatism (EULAR) response criteria at 6 months in the Spanish rheumatoid arthritis (RA) patients. Table S2. Association analysis of the four analyzed genetic variants with the European League Against Rheumatism (EULAR) response criteria at 12 months in the Spanish rheumatoid arthritis (RA) patients. Table S3. Meta-analysis of the four tested genetic variants in non-responder and responder rheumatoid arthritis (RA) patients from the two Spanish collections. [file ar4504-S1.doc]

**ADDITIONAL FILE 1**

**Table S1**. Association analysis of the four analyzed genetic variants with the EULAR response at 6 months in the Spanish RA patients.

|  |  | **6 months** | | | | | | | | |  |
| --- | --- | --- | --- | --- | --- | --- | --- | --- | --- | --- | --- |
|  |  | **Collection 1 (n=438)** | | | |  | **Collection 2 (n=196)** | | | |  |
| **SNP** | **1/2** | **Subgroup (N)** | **MAF** | ***P*-value^a,b^** | **OR [CI 95%]^b^** |  | **Subgroup (N)** | **MAF** | ***P*-value^a,b^** | **OR [CI 95%]^b^** |  |
| rs12081765 | A/G | Responders (n=336) | 0.412 |  |  |  | Responders (n=162) | 0.444 |  |  |  |
|  |  | Non-responders (n=82) | 0.427 | 0.851 | 1.04 [0.71-1.52] |  | Non-responders (n=29) | 0.534 | 0.153 | 1.52 [0.85-2.71] |  |
| rs1532269 | C/G | Responders (n=337) | 0.378 |  |  |  | Responders (n=156) | 0.420 |  |  |  |
|  |  | Non-responders (n=82) | 0.360 | 0.901 | 0.98 [0.67-1.43] |  | Non-responders (n=28) | 0.411 | 0.952 | 1.02 [0.57-1.82] |  |
| rs17301249 | C/G | Responders (n=334) | 0.121 |  |  |  | Responders (n=161) | 0.130 |  |  |  |
|  |  | Non-responders (n=82) | 0.122 | 0.902 | 1.03 [0.59-1.82] |  | Non-responders (n=28) | 0.143 | 0.848 | 1.09 [0.46-2.60] |  |
| rs7305646 | T/C | Responders (n=336) | 0.488 |  |  |  | Responders (n=160) | 0.478 |  |  |  |
|  |  | Non-responders (n=82) | 0.415 | 0.150 | 0.75 [0.50-1.11] |  | Non-responders (n=28) | 0.536 | 0.148 | 1.61 [0.84-3.05] |  |

^a^ All *P*-values have been calculated for the allelic model. ^b^ Adjusted for DAS28 at baseline, gender and anti-TNF treatment.

1= minor allele, 2= major allele

**Table S2**. Association analysis of the four analyzed genetic variants with the EULAR response at 12 months in the Spanish RA patients.

|  |  | **12 months** | | | | | | | | |
| --- | --- | --- | --- | --- | --- | --- | --- | --- | --- | --- |
|  |  | **Collection 1 (n=314)** | | | |  | **Collection 2 (n=131)** | | | |
| **SNP** | **1/2** | **Subgroup (N)** | **MAF** | ***P*-value^a,b^** | **OR [CI 95%]^b^** |  | **Subgroup (N)** | **MAF** | ***P*-value^a,b^** | **OR [CI 95%]^b^** |
| rs12081765 | A/G | Responders (n=259) | 0.398 |  |  |  | Responders (n=114) | 0.395 |  |  |
|  |  | Non-responders (n=55) | 0.473 | 0.226 | 1.31 [0.84-2.04] |  | Non-responders (n=16) | 0.500 | 0.255 | 1.54 [0.73-3.22] |
| rs1532269 | C/G | Responders (n=258) | 0.396 |  |  |  | Responders (n=114) | 0.399 |  |  |
|  |  | Non-responders (n=55) | 0.418 | 0.593 | 1.12 [0.73-1.71] |  | Non-responders (n=16) | 0.563 | 0.114 | 1.78 [0.87-3.64] |
| rs17301249 | C/G | Responders (n=259) | 0.134 |  |  |  | Responders (n=114) | 0.162 |  |  |
|  |  | Non-responders (n=55) | 0.118 | 0.536 | 0.81 [0.42-1.58] |  | Non-responders (n=16) | 0.094 | 0.309 | 0.52 [0.14-1.84] |
| rs7305646 | T/C | Responders (n=259) | 0.494 |  |  |  | Responders (n=113) | 0.469 |  |  |
|  |  | Non-responders (n=55) | 0.445 | 0.43 | 0.82 [0.52-1.33] |  | Non-responders (n=16) | 0.500 | 0.76 | 1.13 [0.52-2.46] |

^a^ All *P*-values have been calculated for the allelic model. ^b^ Adjusted for DAS28 at baseline, gender and anti-TNF treatment.

1= minor allele, 2= major allele

**Table S3**. Meta-analysis of the four tested genetic variants in non-responder and responder RA patients from the two Spanish collections.

|  |  |  | **6 months** | | | |  | **12 months** | | | |
| --- | --- | --- | --- | --- | --- | --- | --- | --- | --- | --- | --- |
| **SNP** | **Locus** | **1/2** | **Subgroup (N)** | **MAF** | ***P*-value*** | **OR [CI 95%]**** |  | **Subgroup (N)** | **MAF (%)** | ***P*-value*** | **OR [CI 95%]**** |
| rs12081765 | *Intergenic* | A/G | Responders (n=498) | 0.423 |  |  |  | Responders (n=373) | 0.397 |  |  |
|  |  |  | Non-responders (n=111) | 0.455 | 0.379 | 1.15 [0.86-1.55] |  | Non-responders (n=71) | 0.479 | 0.084 | 1.40 [0.97-2.00] |
| rs1532269 | *PDZ2D* | C/G | Responders (n=493) | 0.392 |  |  |  | Responders (n=372) | 0.397 |  |  |
|  |  |  | Non-responders (n=110) | 0.373 | 0.715 | 0.93 [0.68-1.28] |  | Non-responders (n=71) | 0.451 | 0.255 | 1.26 [0.86-1.83] |
| rs17301249 | *EYA4* | C/G | Responders (n=495) | 0.124 |  |  |  | Responders (n=372) | 0.143 |  |  |
|  |  |  | Non-responders (n=110) | 0.127 | 0.967 | 1.04 [0.67-1.61] |  | Non-responders (n=71) | 0.113 | 0.449 | 0.78 [0.44-1.36] |
| rs7305646 | *Intergenic* | T/C | Responders (n=496) | 0.485 |  |  |  | Responders (n=372) | 0.487 |  |  |
|  |  |  | Non-responders (n=110) | 0.446 | 0.336 | 0.86 [0.64-1.15] |  | Non-responders (n=71) | 0.458 | 0.575 | 0.89 [0.62-1.27] |
